# Poly[[bis­[μ_2_-1,2-bis­(pyridin-4-yl)ethene-κ^2^ *N*:*N*′]bis­(thio­cyanato-κ*N*)cobalt(II)] 1,2-bis­(pyridin-4-yl)ethene monosolvate dihydrate]

**DOI:** 10.1107/S1600536813021107

**Published:** 2013-08-10

**Authors:** Susanne Wöhlert, Inke Jess, Christian Näther

**Affiliations:** a Institut für Anorganische Chemie, Christian-Albrechts-Universität Kiel, Max-Eyth-Strasse 2, 24118 Kiel, Germany

## Abstract

The asymmetric unit of the title compound, {[Co(NCS)_2_(C_12_H_10_N_2_)_2_]·C_12_H_10_N_2_·2H_2_O}_*n*_, consists of two independent Co^II^ cations, four distinct thio­cyanate anions, six 1,2-bis­(pyridin-4-yl)ethene (4-bpe) mol­ecules and four lattice water mol­ecules. The Co^II^ cations are each coordinated by two *N*-bonded thio­cyanate anions and four 4-bpe ligands within a slightly distorted CoN_6_ octa­hedron. The two independent Co^II^ cations are linked by the 4-bpe ligands into two distinct layers, parallel to the *ac* and *bc* planes, that inter­penetrate. From this arrangement, channels are formed in which non-coordinated 4-bpe and lattice water mol­ecules are hydrogen-bonded into chains that elongate in the *c*-axis direction.

## Related literature

For the background to this work, see: Wöhlert *et al.* (2011 ▶).
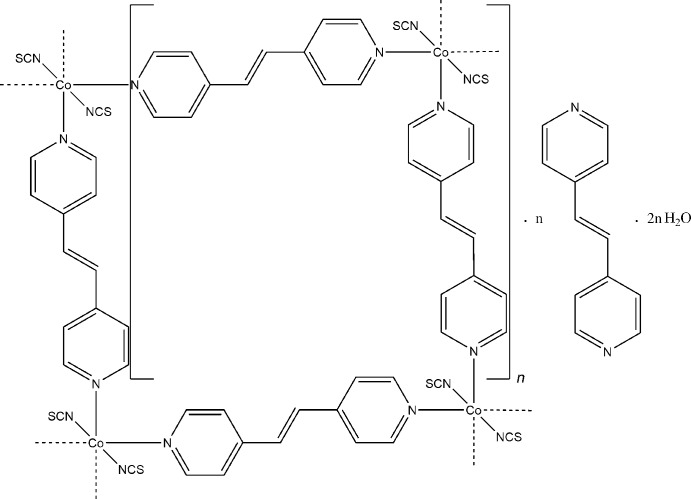


## Experimental

### 

#### Crystal data

[Co(NCS)_2_(C_12_H_10_N_2_)_2_]·C_12_H_10_N_2_·2H_2_O
*M* *_r_* = 757.78
Orthorhombic, 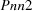

*a* = 22.3698 (11) Å
*b* = 22.4296 (16) Å
*c* = 15.3707 (7) Å
*V* = 7712.2 (8) Å^3^
*Z* = 8
Mo *K*α radiation
μ = 0.60 mm^−1^
*T* = 170 K
0.09 × 0.05 × 0.03 mm

#### Data collection

Stoe IPDS-1 diffractometer
Absorption correction: numerical (*X-SHAPE* and *X-RED32*; Stoe, 2008 ▶) *T*_min_ = 0.849, *T*_max_ = 0.971
34972 measured reflections
9556 independent reflections
7608 reflections with *I* > 2σ(*I*)
*R*_int_ = 0.054
θ_max_ = 22.4°

#### Refinement

*R*[*F* ^2^ > 2σ(*F* ^2^)] = 0.046
*wR*(*F* ^2^) = 0.108
*S* = 1.04
9556 reflections
919 parameters
1 restraint
H-atom parameters constrained
Δρ_max_ = 0.23 e Å^−3^
Δρ_min_ = −0.35 e Å^−3^
Absolute structure: Flack (1983 ▶), 4369 Friedel pairs
Absolute structure parameter: 0.06 (2)

### 

Data collection: *X-AREA* (Stoe, 2008 ▶); cell refinement: *X-AREA*; data reduction: *X-AREA*; program(s) used to solve structure: *SHELXS97* (Sheldrick, 2008 ▶); program(s) used to refine structure: *SHELXL97* (Sheldrick, 2008 ▶); molecular graphics: *SHELXTL* (Sheldrick, 2008 ▶) and *DIAMOND* (Brandenburg, 2011 ▶); software used to prepare material for publication: *SHELXTL*.

## Supplementary Material

Crystal structure: contains datablock(s) I, global. DOI: 10.1107/S1600536813021107/is5295sup1.cif

Structure factors: contains datablock(s) I. DOI: 10.1107/S1600536813021107/is5295Isup2.hkl

Additional supplementary materials:  crystallographic information; 3D view; checkCIF report

## Figures and Tables

**Table 1:** Hydrogen-bond geometry (Å, °)

*D*—H⋯*A*	*D*—H	H⋯*A*	*D*⋯*A*	*D*—H⋯*A*
O1—H2*O*1⋯O3	0.84	2.00	2.825 (8)	169
O2—H2*O*2⋯O4	0.84	2.09	2.818 (8)	144
O3—H2*O*3⋯N91	0.84	2.15	2.933 (9)	156
O4—H1*O*4⋯N110	0.84	2.14	2.885 (9)	148
O1—H1*O*1⋯N90^i^	0.84	2.07	2.877 (10)	160
O2—H1*O*2⋯N111^ii^	0.84	2.03	2.858 (10)	170
O4—H2*O*4⋯O2^iii^	0.84	2.10	2.844 (8)	147

Symmetry codes: (i) 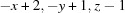; (ii) 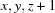; (iii) 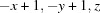.

**Fig. 1.**
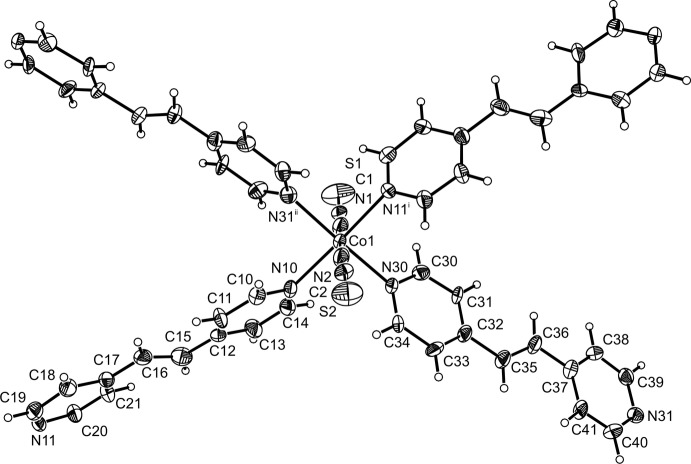
Environment of Co1 with labeling and displacement ellipsoids drawn at the 50% probability level. [Symmetry codes: (i) x - 1/2, -y + 1/2, z + 1/2; (ii) x + 1/2, - y + 1/2, z + 1/2.]

**Fig. 2.**
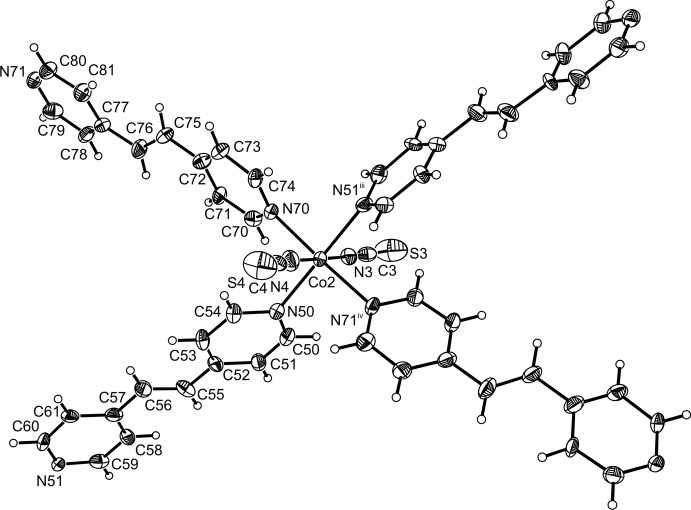
Environment of Co2 with labeling and displacement ellipsoids drawn at the 50% probability level. [Symmetry codes: (iii) -x - 3/2, y - 1/2, z - 1/2; (iv) -x + 3/2, y - 1/2, z + 1/2.]

**Fig. 3.**
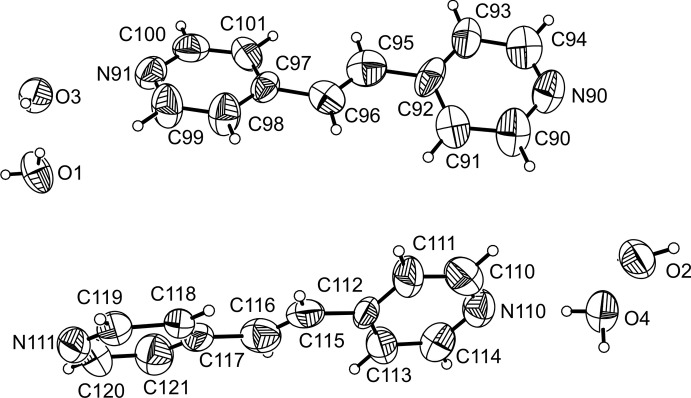
View of the non-coordinated 1,2-bis(pyridin-4-yl)ethene ligands and the water molecules with labeling and displacement ellipsoids drawn at the 50% probability level.

**Fig. 4.**
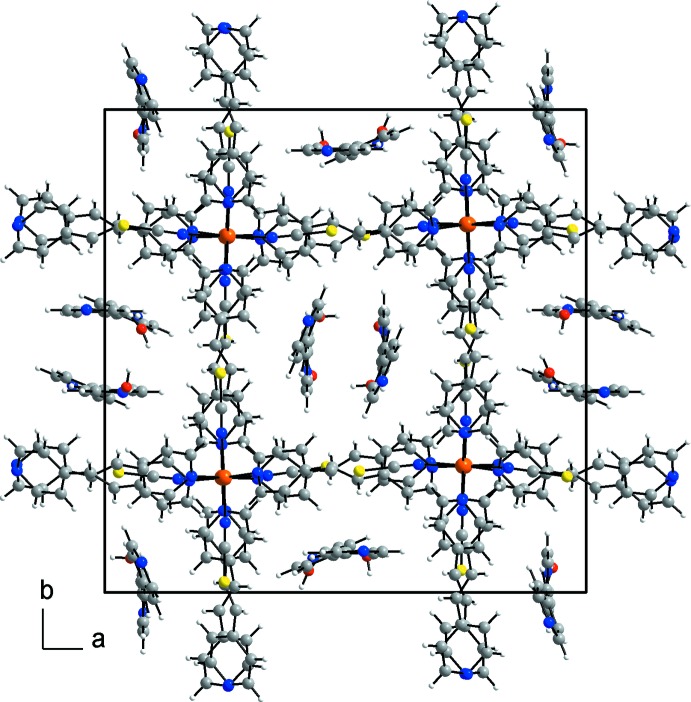
Crystal structure of the title compound with view along the c-axis (orange = cobalt, blue = nitrogen, yellow = sulfur, grey = carbon, white = hydrogen).

## supplementary crystallographic information

### 1. Comment

In our ongoing project on the synthesis, structures and properties of coordination polymers based on transition metal(II) thio- and selenocyanates we investigated compounds with 1,2-bis(pyridin-4-yl)ethene (4-bpe) as co-ligand (Wöhlert *et al.*, 2011). In the course of these investigations crystals of the title compound were obtained that were characterized by single-crystal X-ray diffraction.

The asymmetric unit of the title compound consists of two crystallographic independent cobalt(II) cations (Co1 and Co2), four distinct thiocyanato anions, six 4-bpe ligands and four water molecules (Figs. 1–3). Each cobalt cation is coordinated by two *N*-bonded thiocyanato anions and four 1,2-bis(4-pyridin-4-yl)ethene (4-bpe) ligands within slightly distorted octahedra. The CoN_6_ distances ranges from 2.067 (4) Å to 2.185 (5) Å with angles around the cobalt(II) cations between 87.46 (16) to 93.77 (18)° and 176.95 (16) to 179.22 (18)°. In the crystal structure the cobalt(II) cations are linked by the 4-bpe ligands into layers. There are two kinds of layers. Those based on Co1 are parallel to the *a/c*-plane, whereas those based on Co2 parallel to the *b/c*-plane. These layers interpenetrate to form channels that elongate along the *c*-axis. Within these channels additional non-coordinating 4-bpe ligands and water molecules are embedded that are hydrogen bonded into chains (Table 1).

### 2. Experimental

Co(NCS)_2_ and 1,2-bis(pyridin-4-yl)ethene were obtained from Alfa Aesar and Sigma Aldrich. All chemicals were used without further purification. 0.15 mmol (28 mg) Co(NCS)_2_ and 0.6 mmol (108 mg) 1,2-bis(pyridin-4-yl)ethene were reacted in 1 ml H_2_O in a closed test-tube at 120 °C for three days. On cooling red block-shaped single crystals of the title compound were obtained.

### 3. Refinement

All H atoms were positions with idealized geometry and were refined isotropic with *U*_iso_(H) = 1.2 *U*_eq_(C) using a riding model with C—H = 0.95 Å. The O—H hydrogen atoms were located in a difference map, their bond lengths were set to ideal values of 0.84 Å and finally they were refined with *U*_iso_(H) = 1.5 *U*_eq_(O) using a riding model. The absolute structure was determined on the basis of 4369 Friedel pairs. Because of the large unit-cell parameters and the relatively large width of the reflection profiles diffraction data were measured to theta of only 22.4°

### Crystal data

**Table:**

[Co(NCS)_2_(C_12_H_10_N_2_)_2_]·C_12_H_10_N_2_·2H_2_O	*F*(000) = 3144
*M**_r_* = 757.78	*D*_x_ = 1.305 Mg m^−^^3^
Orthorhombic, *P**n**n*2	Mo *K*α radiation, λ = 0.71073 Å
Hall symbol: P 2 -2n	Cell parameters from 34972 reflections
*a* = 22.3698 (11) Å	θ = 1.6–22.4°
*b* = 22.4296 (16) Å	µ = 0.60 mm^−^^1^
*c* = 15.3707 (7) Å	*T* = 170 K
*V* = 7712.2 (8) Å^3^	Block, red
*Z* = 8	0.09 × 0.05 × 0.03 mm

### Data collection

**Table:**

Stoe IPDS-1 diffractometer	9556 independent reflections
Radiation source: fine-focus sealed tube	7608 reflections with *I* > 2σ(*I*)
Graphite monochromator	*R*_int_ = 0.054
φ scan	θ_max_ = 22.4°, θ_min_ = 1.6°
Absorption correction: numerical (*X-SHAPE* and *X-RED32*; Stoe, 2008)	*h* = −23→24
*T*_min_ = 0.849, *T*_max_ = 0.971	*k* = −24→24
34972 measured reflections	*l* = −15→16

### Refinement

**Table:**

Refinement on *F*^2^	Secondary atom site location: difference Fourier map
Least-squares matrix: full	Hydrogen site location: inferred from neighbouring sites
*R*[*F*^2^ > 2σ(*F*^2^)] = 0.046	H-atom parameters constrained
*wR*(*F*^2^) = 0.108	*w* = 1/[σ^2^(*F*_o_^2^) + (0.0477*P*)^2^ + 5.5114*P*] where *P* = (*F*_o_^2^ + 2*F*_c_^2^)/3
*S* = 1.04	(Δ/σ)_max_ = 0.001
9556 reflections	Δρ_max_ = 0.23 e Å^−^^3^
919 parameters	Δρ_min_ = −0.35 e Å^−^^3^
1 restraint	Absolute structure: Flack (1983), 4369 Friedel pairs
Primary atom site location: structure-invariant direct methods	Absolute structure parameter: 0.06 (2)

### Special details

**Table:**

Geometry. All e.s.d.'s (except the e.s.d. in the dihedral angle between two l.s. planes) are estimated using the full covariance matrix. The cell e.s.d.'s are taken into account individually in the estimation of e.s.d.'s in distances, angles and torsion angles; correlations between e.s.d.'s in cell parameters are only used when they are defined by crystal symmetry. An approximate (isotropic) treatment of cell e.s.d.'s is used for estimating e.s.d.'s involving l.s. planes.
Refinement. Refinement of *F*^2^ against ALL reflections. The weighted *R*-factor *wR* and goodness of fit *S* are based on *F*^2^, conventional *R*-factors *R* are based on *F*, with *F* set to zero for negative *F*^2^. The threshold expression of *F*^2^ > σ(*F*^2^) is used only for calculating *R*-factors(gt) *etc*. and is not relevant to the choice of reflections for refinement. *R*-factors based on *F*^2^ are statistically about twice as large as those based on *F*, and *R*- factors based on ALL data will be even larger.

### Fractional atomic coordinates and isotropic or equivalent isotropic displacement parameters (Å^2^)

**Table:**

	*x*	*y*	*z*	*U*_iso_*/*U*_eq_	
Co1	0.74806 (2)	0.26235 (3)	1.03062 (6)	0.02261 (17)	
Co2	0.75553 (3)	−0.23789 (2)	1.04067 (5)	0.02213 (17)	
N1	0.74285 (18)	0.1698 (2)	1.0203 (3)	0.0334 (12)	
C1	0.7421 (2)	0.1186 (3)	1.0282 (4)	0.0347 (13)	
S1	0.74027 (11)	0.04599 (6)	1.03888 (18)	0.0737 (6)	
N2	0.75022 (18)	0.35535 (18)	1.0409 (4)	0.0316 (9)	
C2	0.7492 (2)	0.40689 (19)	1.0366 (4)	0.0254 (10)	
S2	0.74847 (9)	0.47821 (6)	1.02980 (16)	0.0560 (4)	
N3	0.66189 (19)	−0.24203 (17)	1.0426 (4)	0.0324 (10)	
C3	0.6104 (2)	−0.2494 (2)	1.0418 (4)	0.0284 (12)	
S3	0.53951 (6)	−0.26009 (11)	1.03899 (18)	0.0754 (6)	
N4	0.84792 (18)	−0.23491 (18)	1.0373 (4)	0.0335 (10)	
C4	0.8991 (2)	−0.2401 (2)	1.0385 (4)	0.0296 (12)	
S4	0.97128 (6)	−0.24874 (8)	1.03817 (17)	0.0605 (5)	
N10	0.8173 (2)	0.2646 (2)	0.9323 (3)	0.0276 (12)	
C10	0.8632 (2)	0.3026 (3)	0.9388 (4)	0.0322 (14)	
H10	0.8602	0.3346	0.9789	0.039*	
C11	0.9143 (2)	0.2974 (3)	0.8904 (4)	0.0330 (15)	
H11	0.9462	0.3248	0.8986	0.040*	
C12	0.9197 (3)	0.2517 (3)	0.8285 (4)	0.0294 (14)	
C13	0.8705 (3)	0.2140 (3)	0.8203 (4)	0.0377 (15)	
H13	0.8714	0.1826	0.7789	0.045*	
C14	0.8213 (2)	0.2215 (3)	0.8708 (4)	0.0331 (14)	
H14	0.7882	0.1955	0.8627	0.040*	
C15	0.9730 (3)	0.2412 (3)	0.7771 (5)	0.0416 (17)	
H15	0.9699	0.2145	0.7294	0.050*	
C16	1.0256 (2)	0.2660 (3)	0.7918 (5)	0.0353 (14)	
H16	1.0292	0.2924	0.8399	0.042*	
C17	1.0791 (2)	0.2553 (3)	0.7377 (4)	0.0288 (13)	
C18	1.1289 (2)	0.2931 (3)	0.7447 (4)	0.0336 (14)	
H18	1.1290	0.3243	0.7865	0.040*	
C19	1.1784 (3)	0.2850 (3)	0.6902 (4)	0.0342 (15)	
H19	1.2111	0.3119	0.6940	0.041*	
C20	1.1344 (2)	0.2022 (2)	0.6300 (4)	0.0311 (14)	
H20	1.1369	0.1695	0.5910	0.037*	
C21	1.0843 (2)	0.2080 (2)	0.6797 (4)	0.0288 (14)	
H21	1.0529	0.1797	0.6747	0.035*	
N11	1.1806 (2)	0.2407 (2)	0.6334 (3)	0.0256 (11)	
N30	0.6761 (2)	0.2686 (2)	0.9370 (3)	0.0265 (12)	
C30	0.6315 (2)	0.2294 (2)	0.9442 (4)	0.0307 (14)	
H30	0.6354	0.1977	0.9849	0.037*	
C31	0.5796 (2)	0.2328 (3)	0.8950 (4)	0.0295 (14)	
H31	0.5484	0.2046	0.9031	0.035*	
C32	0.5738 (2)	0.2772 (3)	0.8344 (4)	0.0279 (13)	
C33	0.6202 (2)	0.3179 (3)	0.8254 (4)	0.0327 (14)	
H33	0.6178	0.3492	0.7840	0.039*	
C34	0.6705 (2)	0.3119 (3)	0.8781 (4)	0.0283 (14)	
H34	0.7021	0.3398	0.8718	0.034*	
C35	0.5200 (2)	0.2831 (2)	0.7799 (4)	0.0321 (13)	
H35	0.5212	0.3104	0.7327	0.038*	
C36	0.4699 (2)	0.2524 (3)	0.7928 (5)	0.0334 (15)	
H36	0.4682	0.2278	0.8431	0.040*	
C37	0.4169 (3)	0.2531 (2)	0.7362 (4)	0.0306 (15)	
C38	0.3736 (2)	0.2096 (2)	0.7459 (4)	0.0324 (13)	
H38	0.3770	0.1809	0.7910	0.039*	
C39	0.3252 (2)	0.2082 (3)	0.6894 (4)	0.0304 (14)	
H39	0.2961	0.1778	0.6967	0.037*	
C40	0.3598 (2)	0.2911 (2)	0.6183 (4)	0.0314 (14)	
H40	0.3549	0.3203	0.5740	0.038*	
C41	0.4089 (2)	0.2953 (2)	0.6713 (4)	0.0297 (14)	
H41	0.4370	0.3266	0.6637	0.036*	
N31	0.3177 (2)	0.2478 (2)	0.6253 (3)	0.0276 (12)	
N50	0.7533 (2)	−0.1692 (2)	1.1388 (3)	0.0286 (12)	
C50	0.7075 (3)	−0.1619 (2)	1.1948 (4)	0.0321 (15)	
H50	0.6790	−0.1931	1.1996	0.039*	
C51	0.6994 (3)	−0.1122 (2)	1.2451 (4)	0.0330 (14)	
H51	0.6659	−0.1094	1.2828	0.040*	
C52	0.7406 (3)	−0.0659 (2)	1.2405 (4)	0.0303 (15)	
C53	0.7884 (3)	−0.0727 (2)	1.1856 (4)	0.0358 (15)	
H53	0.8180	−0.0424	1.1816	0.043*	
C54	0.7935 (3)	−0.1243 (2)	1.1358 (4)	0.0310 (14)	
H54	0.8268	−0.1281	1.0979	0.037*	
C55	0.7300 (3)	−0.0120 (2)	1.2907 (5)	0.0346 (15)	
H55	0.6979	−0.0124	1.3312	0.042*	
C56	0.7619 (3)	0.0377 (2)	1.2841 (5)	0.0319 (13)	
H56	0.7928	0.0384	1.2418	0.038*	
C57	0.7533 (3)	0.0918 (3)	1.3371 (4)	0.0278 (14)	
C58	0.7057 (2)	0.0992 (2)	1.3930 (4)	0.0279 (13)	
H58	0.6755	0.0696	1.3965	0.033*	
C59	0.7020 (3)	0.1506 (2)	1.4447 (4)	0.0313 (14)	
H59	0.6692	0.1548	1.4834	0.038*	
C60	0.7886 (3)	0.1875 (2)	1.3839 (4)	0.0309 (14)	
H60	0.8178	0.2182	1.3799	0.037*	
C61	0.7942 (3)	0.1382 (2)	1.3309 (4)	0.0295 (13)	
H61	0.8260	0.1360	1.2901	0.035*	
N51	0.7432 (2)	0.1940 (2)	1.4414 (3)	0.0253 (11)	
N70	0.7484 (2)	−0.1683 (2)	0.9427 (3)	0.0250 (11)	
C70	0.7095 (3)	−0.1230 (2)	0.9541 (4)	0.0311 (14)	
H70	0.6815	−0.1258	1.0003	0.037*	
C71	0.7083 (2)	−0.0737 (2)	0.9029 (4)	0.0267 (13)	
H71	0.6808	−0.0424	0.9146	0.032*	
C72	0.7477 (3)	−0.0694 (3)	0.8331 (4)	0.0336 (16)	
C73	0.7863 (3)	−0.1166 (3)	0.8190 (4)	0.0339 (15)	
H73	0.8129	−0.1162	0.7709	0.041*	
C74	0.7859 (3)	−0.1653 (2)	0.8765 (4)	0.0334 (15)	
H74	0.8133	−0.1970	0.8675	0.040*	
C75	0.7502 (3)	−0.0165 (2)	0.7759 (5)	0.0321 (16)	
H75	0.7690	−0.0208	0.7209	0.039*	
C76	0.7277 (3)	0.0368 (2)	0.7965 (5)	0.0361 (15)	
H76	0.7067	0.0405	0.8500	0.043*	
C77	0.7334 (3)	0.0901 (3)	0.7416 (4)	0.0278 (14)	
C78	0.6960 (3)	0.1386 (2)	0.7543 (4)	0.0341 (15)	
H78	0.6670	0.1383	0.7995	0.041*	
C79	0.7017 (3)	0.1882 (3)	0.6995 (4)	0.0348 (15)	
H79	0.6755	0.2211	0.7078	0.042*	
C80	0.7796 (2)	0.1444 (2)	0.6259 (4)	0.0294 (14)	
H80	0.8092	0.1464	0.5817	0.035*	
C81	0.7763 (3)	0.0937 (2)	0.6770 (4)	0.0314 (14)	
H81	0.8033	0.0617	0.6678	0.038*	
N71	0.7421 (2)	0.19110 (19)	0.6365 (3)	0.0274 (12)	
N90	0.9622 (4)	0.5853 (3)	0.6652 (5)	0.078 (2)	
C90	0.9232 (5)	0.5839 (4)	0.5997 (7)	0.083 (3)	
H90	0.8816	0.5849	0.6124	0.100*	
C91	0.9417 (4)	0.5811 (3)	0.5125 (6)	0.071 (2)	
H91	0.9131	0.5788	0.4669	0.085*	
C92	1.0029 (4)	0.5817 (3)	0.4937 (6)	0.070 (3)	
C93	1.0415 (5)	0.5825 (4)	0.5614 (6)	0.093 (3)	
H93	1.0834	0.5823	0.5517	0.112*	
C94	1.0188 (5)	0.5837 (5)	0.6452 (7)	0.102 (3)	
H94	1.0467	0.5834	0.6918	0.123*	
C95	1.0271 (4)	0.5785 (4)	0.4014 (7)	0.078 (3)	
H95	1.0690	0.5727	0.3957	0.094*	
C96	0.9982 (4)	0.5825 (3)	0.3325 (6)	0.064 (2)	
H96	0.9564	0.5890	0.3369	0.077*	
C97	1.0247 (3)	0.5778 (3)	0.2423 (5)	0.0526 (18)	
C98	0.9905 (4)	0.5977 (4)	0.1744 (6)	0.078 (2)	
H98	0.9521	0.6145	0.1842	0.093*	
C99	1.0135 (4)	0.5925 (4)	0.0903 (6)	0.083 (3)	
H99	0.9891	0.6054	0.0434	0.100*	
C100	1.0998 (4)	0.5517 (4)	0.1381 (7)	0.069 (2)	
H100	1.1382	0.5353	0.1265	0.083*	
C101	1.0804 (4)	0.5545 (3)	0.2233 (6)	0.066 (2)	
H101	1.1054	0.5405	0.2689	0.079*	
N91	1.0670 (3)	0.5709 (3)	0.0715 (5)	0.0666 (18)	
N110	0.5768 (3)	0.4377 (3)	0.4991 (5)	0.0718 (19)	
C110	0.5986 (4)	0.4907 (5)	0.4782 (7)	0.092 (3)	
H110	0.6131	0.5151	0.5241	0.111*	
C111	0.6016 (4)	0.5132 (4)	0.3949 (6)	0.082 (3)	
H111	0.6183	0.5515	0.3848	0.098*	
C112	0.5807 (3)	0.4802 (4)	0.3272 (5)	0.059 (2)	
C113	0.5584 (4)	0.4241 (4)	0.3453 (6)	0.071 (2)	
H113	0.5443	0.3993	0.2996	0.085*	
C114	0.5566 (4)	0.4041 (4)	0.4320 (6)	0.073 (2)	
H114	0.5406	0.3657	0.4439	0.087*	
C115	0.5834 (3)	0.5061 (4)	0.2376 (7)	0.072 (2)	
H115	0.5910	0.5476	0.2331	0.087*	
C116	0.5764 (4)	0.4776 (4)	0.1677 (7)	0.076 (3)	
H116	0.5684	0.4362	0.1733	0.091*	
C117	0.5792 (3)	0.5015 (4)	0.0763 (6)	0.068 (2)	
C118	0.5797 (3)	0.5619 (4)	0.0584 (5)	0.068 (2)	
H118	0.5797	0.5903	0.1042	0.081*	
C119	0.5803 (4)	0.5808 (4)	−0.0309 (6)	0.072 (2)	
H119	0.5798	0.6223	−0.0432	0.087*	
C120	0.5804 (4)	0.4843 (5)	−0.0766 (6)	0.079 (3)	
H120	0.5807	0.4562	−0.1228	0.095*	
C121	0.5790 (4)	0.4635 (4)	0.0068 (7)	0.084 (3)	
H121	0.5778	0.4217	0.0168	0.101*	
N111	0.5815 (3)	0.5428 (3)	−0.0968 (5)	0.0731 (19)	
O1	1.0450 (3)	0.4249 (2)	−0.1485 (4)	0.0807 (17)	
H1O1	1.0516	0.4188	−0.2015	0.121*	
H2O1	1.0506	0.4605	−0.1335	0.121*	
O2	0.5698 (2)	0.5514 (2)	0.7185 (4)	0.0801 (17)	
H1O2	0.5740	0.5533	0.7727	0.120*	
H2O2	0.5815	0.5165	0.7081	0.120*	
O3	1.0756 (3)	0.5449 (3)	−0.1151 (4)	0.0800 (18)	
H1O3	1.1130	0.5481	−0.1165	0.120*	
H2O3	1.0621	0.5513	−0.0650	0.120*	
O4	0.5535 (3)	0.4292 (2)	0.6832 (4)	0.0746 (17)	
H1O4	0.5630	0.4170	0.6335	0.112*	
H2O4	0.5165	0.4271	0.6743	0.112*	

### Atomic displacement parameters (Å^2^)

**Table:**

	*U*^11^	*U*^22^	*U*^33^	*U*^12^	*U*^13^	*U*^23^
Co1	0.0163 (3)	0.0301 (3)	0.0215 (4)	0.0034 (3)	−0.0018 (4)	0.0021 (4)
Co2	0.0295 (3)	0.0160 (3)	0.0209 (4)	−0.0024 (3)	−0.0006 (4)	0.0022 (4)
N1	0.026 (2)	0.039 (3)	0.035 (3)	0.007 (2)	−0.003 (2)	0.000 (2)
C1	0.027 (3)	0.058 (4)	0.019 (3)	0.007 (3)	−0.002 (3)	0.002 (3)
S1	0.1349 (17)	0.0268 (8)	0.0595 (11)	−0.0010 (10)	−0.0109 (15)	0.0057 (11)
N2	0.0234 (19)	0.041 (3)	0.031 (3)	0.008 (2)	−0.001 (3)	−0.002 (3)
C2	0.032 (2)	0.020 (3)	0.024 (3)	0.010 (2)	−0.002 (3)	−0.002 (3)
S2	0.0806 (10)	0.0353 (8)	0.0520 (11)	0.0042 (8)	−0.0020 (11)	0.0065 (10)
N3	0.047 (3)	0.022 (2)	0.028 (3)	−0.0009 (19)	−0.004 (3)	−0.001 (2)
C3	0.028 (3)	0.035 (3)	0.022 (3)	0.005 (2)	0.004 (3)	−0.002 (3)
S3	0.0335 (8)	0.1385 (17)	0.0543 (13)	−0.0099 (10)	−0.0016 (12)	−0.0021 (14)
N4	0.032 (2)	0.031 (2)	0.038 (3)	−0.0063 (19)	−0.007 (3)	0.001 (2)
C4	0.044 (3)	0.024 (3)	0.021 (3)	−0.002 (2)	−0.005 (4)	−0.004 (3)
S4	0.0302 (7)	0.0890 (13)	0.0623 (12)	0.0098 (7)	−0.0049 (12)	−0.0077 (12)
N10	0.017 (3)	0.037 (3)	0.029 (3)	0.000 (2)	−0.002 (2)	−0.001 (2)
C10	0.027 (3)	0.038 (3)	0.032 (4)	0.002 (3)	−0.002 (3)	0.001 (3)
C11	0.018 (3)	0.042 (4)	0.039 (4)	−0.004 (3)	0.007 (3)	0.000 (3)
C12	0.024 (4)	0.050 (4)	0.014 (3)	−0.001 (3)	0.000 (3)	0.002 (3)
C13	0.032 (3)	0.051 (4)	0.030 (4)	−0.001 (3)	0.006 (3)	−0.008 (3)
C14	0.026 (3)	0.045 (4)	0.029 (4)	−0.008 (3)	−0.001 (3)	0.000 (3)
C15	0.047 (4)	0.051 (4)	0.027 (4)	0.002 (3)	0.003 (4)	−0.007 (3)
C16	0.032 (3)	0.050 (4)	0.024 (3)	0.010 (3)	0.004 (3)	0.004 (3)
C17	0.024 (3)	0.035 (3)	0.027 (4)	0.006 (3)	0.003 (3)	0.005 (3)
C18	0.030 (3)	0.047 (4)	0.024 (3)	0.004 (3)	0.003 (3)	−0.007 (3)
C19	0.031 (3)	0.043 (4)	0.029 (4)	−0.006 (3)	−0.006 (3)	−0.004 (3)
C20	0.025 (3)	0.035 (3)	0.034 (4)	−0.005 (3)	0.003 (3)	−0.004 (3)
C21	0.022 (3)	0.024 (3)	0.041 (4)	−0.002 (2)	0.008 (3)	−0.003 (3)
N11	0.021 (3)	0.033 (3)	0.022 (3)	−0.003 (2)	0.004 (2)	−0.002 (2)
N30	0.016 (3)	0.040 (3)	0.024 (3)	0.000 (2)	−0.003 (2)	0.002 (2)
C30	0.033 (3)	0.029 (3)	0.030 (4)	0.005 (3)	0.002 (3)	0.009 (3)
C31	0.012 (3)	0.044 (4)	0.032 (4)	0.003 (2)	−0.007 (2)	0.002 (3)
C32	0.020 (3)	0.039 (3)	0.025 (3)	−0.001 (3)	−0.008 (3)	−0.004 (3)
C33	0.031 (3)	0.044 (4)	0.023 (3)	−0.002 (3)	−0.011 (3)	0.008 (3)
C34	0.012 (3)	0.041 (3)	0.031 (4)	−0.002 (2)	0.000 (2)	0.005 (3)
C35	0.023 (3)	0.044 (3)	0.029 (3)	0.000 (2)	−0.010 (3)	−0.002 (3)
C36	0.022 (3)	0.043 (4)	0.035 (4)	0.006 (2)	−0.009 (3)	0.001 (3)
C37	0.025 (4)	0.031 (3)	0.036 (4)	0.004 (3)	−0.005 (3)	−0.001 (3)
C38	0.032 (3)	0.044 (4)	0.021 (3)	0.002 (3)	−0.002 (3)	0.005 (3)
C39	0.025 (3)	0.031 (3)	0.036 (4)	−0.004 (2)	0.003 (3)	0.003 (3)
C40	0.039 (3)	0.027 (3)	0.028 (4)	−0.002 (3)	−0.007 (3)	0.006 (3)
C41	0.017 (3)	0.034 (3)	0.039 (4)	−0.009 (2)	−0.011 (3)	0.001 (3)
N31	0.028 (3)	0.026 (3)	0.028 (3)	−0.004 (2)	0.002 (2)	0.001 (2)
N50	0.039 (3)	0.023 (3)	0.024 (3)	−0.006 (2)	−0.001 (2)	0.003 (2)
C50	0.041 (4)	0.026 (3)	0.029 (4)	−0.009 (3)	0.005 (3)	0.005 (3)
C51	0.044 (3)	0.027 (3)	0.028 (3)	−0.012 (3)	0.010 (3)	−0.005 (3)
C52	0.053 (4)	0.014 (3)	0.024 (3)	0.001 (3)	−0.001 (3)	−0.008 (3)
C53	0.051 (4)	0.016 (3)	0.041 (4)	−0.006 (3)	0.001 (3)	−0.001 (3)
C54	0.036 (3)	0.022 (3)	0.035 (4)	−0.005 (2)	0.001 (3)	0.002 (3)
C55	0.041 (4)	0.034 (4)	0.029 (3)	−0.001 (3)	−0.003 (4)	−0.012 (3)
C56	0.042 (3)	0.028 (3)	0.026 (3)	−0.003 (3)	0.008 (3)	−0.007 (3)
C57	0.038 (4)	0.028 (3)	0.018 (3)	0.003 (3)	0.001 (3)	−0.002 (2)
C58	0.038 (3)	0.019 (3)	0.027 (4)	0.000 (2)	−0.001 (3)	−0.006 (2)
C59	0.038 (3)	0.034 (4)	0.022 (4)	0.008 (3)	0.000 (3)	−0.001 (3)
C60	0.039 (3)	0.020 (3)	0.033 (4)	−0.008 (2)	0.003 (3)	0.007 (3)
C61	0.046 (3)	0.028 (3)	0.015 (3)	0.000 (3)	0.003 (3)	−0.005 (3)
N51	0.036 (3)	0.021 (3)	0.019 (3)	−0.006 (2)	−0.001 (2)	0.0029 (19)
N70	0.033 (3)	0.020 (3)	0.023 (3)	−0.004 (2)	0.002 (2)	0.0025 (19)
C70	0.037 (3)	0.032 (4)	0.024 (4)	−0.003 (3)	0.003 (3)	−0.003 (3)
C71	0.035 (3)	0.014 (3)	0.031 (4)	0.004 (2)	−0.002 (3)	0.007 (2)
C72	0.044 (4)	0.033 (4)	0.023 (3)	−0.003 (3)	−0.005 (3)	0.008 (3)
C73	0.044 (3)	0.034 (3)	0.024 (4)	−0.007 (3)	0.008 (3)	0.006 (2)
C74	0.053 (4)	0.014 (3)	0.033 (4)	−0.001 (3)	0.002 (3)	0.003 (3)
C75	0.044 (3)	0.030 (3)	0.022 (4)	−0.005 (3)	0.000 (3)	0.010 (3)
C76	0.049 (4)	0.023 (3)	0.036 (4)	−0.005 (3)	−0.005 (3)	0.011 (3)
C77	0.034 (3)	0.029 (4)	0.020 (3)	−0.005 (3)	0.000 (3)	0.004 (3)
C78	0.047 (4)	0.027 (3)	0.028 (4)	−0.004 (3)	0.011 (3)	0.005 (3)
C79	0.038 (3)	0.038 (4)	0.029 (4)	0.008 (3)	−0.004 (3)	0.003 (3)
C80	0.031 (3)	0.027 (3)	0.030 (4)	0.006 (2)	0.009 (3)	0.005 (3)
C81	0.038 (3)	0.024 (3)	0.032 (4)	0.005 (2)	−0.003 (3)	0.005 (3)
N71	0.037 (3)	0.018 (3)	0.027 (3)	0.000 (2)	−0.002 (2)	0.007 (2)
N90	0.087 (5)	0.086 (5)	0.060 (5)	0.020 (4)	0.014 (5)	0.016 (4)
C90	0.097 (7)	0.086 (6)	0.067 (7)	0.042 (5)	0.010 (6)	0.009 (5)
C91	0.095 (6)	0.056 (5)	0.062 (6)	0.022 (4)	−0.002 (5)	0.003 (4)
C92	0.103 (7)	0.040 (4)	0.068 (6)	−0.011 (4)	0.038 (5)	0.012 (4)
C93	0.091 (7)	0.135 (9)	0.054 (7)	−0.007 (6)	0.011 (6)	0.003 (5)
C94	0.109 (9)	0.122 (9)	0.075 (8)	−0.005 (7)	0.007 (7)	−0.002 (6)
C95	0.082 (7)	0.058 (5)	0.095 (8)	0.001 (5)	−0.004 (6)	0.011 (5)
C96	0.072 (5)	0.049 (5)	0.072 (6)	0.003 (4)	−0.015 (5)	0.001 (4)
C97	0.064 (5)	0.042 (4)	0.052 (5)	−0.008 (3)	0.013 (4)	−0.002 (3)
C98	0.083 (6)	0.088 (6)	0.062 (7)	0.021 (5)	0.011 (5)	0.004 (5)
C99	0.088 (7)	0.104 (7)	0.057 (7)	0.026 (5)	−0.004 (5)	0.021 (5)
C100	0.059 (5)	0.064 (5)	0.085 (7)	−0.015 (4)	−0.002 (5)	−0.012 (5)
C101	0.078 (6)	0.066 (5)	0.055 (6)	−0.002 (4)	−0.016 (5)	−0.004 (4)
N91	0.066 (4)	0.073 (4)	0.060 (5)	−0.007 (3)	0.015 (4)	−0.006 (3)
N110	0.078 (5)	0.080 (5)	0.058 (5)	0.008 (4)	0.005 (4)	0.011 (4)
C110	0.092 (7)	0.111 (8)	0.074 (8)	−0.010 (6)	−0.017 (6)	0.004 (6)
C111	0.098 (7)	0.097 (7)	0.050 (6)	−0.013 (5)	0.005 (5)	0.017 (5)
C112	0.043 (4)	0.094 (6)	0.041 (5)	0.008 (4)	0.011 (4)	0.011 (5)
C113	0.076 (6)	0.083 (6)	0.055 (6)	0.009 (5)	−0.004 (5)	−0.004 (5)
C114	0.080 (6)	0.063 (5)	0.076 (7)	0.010 (4)	0.013 (5)	0.012 (5)
C115	0.043 (4)	0.094 (6)	0.080 (7)	−0.006 (4)	0.000 (5)	−0.011 (6)
C116	0.063 (6)	0.077 (6)	0.088 (8)	0.005 (5)	−0.011 (5)	0.007 (6)
C117	0.042 (4)	0.100 (7)	0.061 (6)	−0.009 (4)	−0.007 (4)	0.032 (5)
C118	0.053 (4)	0.103 (7)	0.047 (6)	−0.029 (4)	−0.002 (4)	0.001 (4)
C119	0.071 (6)	0.084 (6)	0.063 (7)	−0.027 (4)	−0.003 (5)	0.006 (5)
C120	0.083 (6)	0.099 (8)	0.055 (6)	−0.003 (5)	−0.016 (5)	0.002 (5)
C121	0.087 (6)	0.089 (7)	0.077 (8)	0.015 (5)	0.000 (5)	0.006 (6)
N111	0.078 (5)	0.082 (5)	0.059 (5)	−0.024 (4)	−0.007 (4)	0.018 (4)
O1	0.119 (5)	0.065 (3)	0.059 (4)	0.021 (3)	−0.018 (3)	−0.010 (3)
O2	0.096 (4)	0.087 (4)	0.057 (4)	−0.026 (3)	−0.008 (3)	0.004 (3)
O3	0.078 (4)	0.102 (5)	0.060 (4)	−0.025 (3)	0.012 (3)	−0.009 (3)
O4	0.085 (4)	0.081 (4)	0.058 (4)	0.027 (3)	0.005 (3)	0.006 (3)

### Geometric parameters (Å, º)

**Table:**

Co1—N1	2.086 (5)	C59—N51	1.341 (7)
Co1—N2	2.093 (4)	C59—H59	0.9500
Co1—N31^i^	2.145 (5)	C60—N51	1.354 (7)
Co1—N30	2.163 (5)	C60—C61	1.380 (8)
Co1—N10	2.165 (5)	C60—H60	0.9500
Co1—N11^ii^	2.185 (5)	C61—H61	0.9500
Co2—N4	2.068 (4)	N51—Co2^vii^	2.159 (5)
Co2—N3	2.097 (4)	N70—C74	1.320 (8)
Co2—N50	2.157 (5)	N70—C70	1.348 (7)
Co2—N51^iii^	2.159 (5)	C70—C71	1.358 (8)
Co2—N71^iv^	2.170 (4)	C70—H70	0.9500
Co2—N70	2.175 (5)	C71—C72	1.393 (8)
N1—C1	1.155 (6)	C71—H71	0.9500
C1—S1	1.637 (6)	C72—C73	1.384 (8)
N2—C2	1.158 (5)	C72—C75	1.476 (9)
C2—S2	1.603 (5)	C73—C74	1.404 (8)
N3—C3	1.163 (6)	C73—H73	0.9500
C3—S3	1.605 (5)	C74—H74	0.9500
N4—C4	1.152 (5)	C75—C76	1.336 (8)
C4—S4	1.626 (5)	C75—H75	0.9500
N10—C10	1.338 (7)	C76—C77	1.468 (8)
N10—C14	1.353 (8)	C76—H76	0.9500
C10—C11	1.368 (8)	C77—C81	1.384 (8)
C10—H10	0.9500	C77—C78	1.388 (8)
C11—C12	1.404 (9)	C78—C79	1.401 (8)
C11—H11	0.9500	C78—H78	0.9500
C12—C13	1.394 (8)	C79—N71	1.327 (8)
C12—C15	1.450 (9)	C79—H79	0.9500
C13—C14	1.357 (8)	C80—N71	1.351 (7)
C13—H13	0.9500	C80—C81	1.383 (8)
C14—H14	0.9500	C80—H80	0.9500
C15—C16	1.322 (8)	C81—H81	0.9500
C15—H15	0.9500	N71—Co2^viii^	2.170 (4)
C16—C17	1.477 (8)	N90—C94	1.304 (12)
C16—H16	0.9500	N90—C90	1.332 (12)
C17—C21	1.392 (8)	C90—C91	1.405 (12)
C17—C18	1.404 (8)	C90—H90	0.9500
C18—C19	1.400 (8)	C91—C92	1.399 (11)
C18—H18	0.9500	C91—H91	0.9500
C19—N11	1.324 (7)	C92—C93	1.352 (12)
C19—H19	0.9500	C92—C95	1.520 (13)
C20—N11	1.347 (7)	C93—C94	1.385 (14)
C20—C21	1.362 (8)	C93—H93	0.9500
C20—H20	0.9500	C94—H94	0.9500
C21—H21	0.9500	C95—C96	1.243 (11)
N11—Co1^v^	2.185 (5)	C95—H95	0.9500
N30—C34	1.334 (7)	C96—C97	1.511 (11)
N30—C30	1.335 (7)	C96—H96	0.9500
C30—C31	1.388 (8)	C97—C98	1.370 (11)
C30—H30	0.9500	C97—C101	1.382 (10)
C31—C32	1.370 (8)	C98—C99	1.396 (12)
C31—H31	0.9500	C98—H98	0.9500
C32—C33	1.390 (8)	C99—N91	1.324 (10)
C32—C35	1.472 (8)	C99—H99	0.9500
C33—C34	1.391 (8)	C100—N91	1.330 (10)
C33—H33	0.9500	C100—C101	1.382 (12)
C34—H34	0.9500	C100—H100	0.9500
C35—C36	1.330 (8)	C101—H101	0.9500
C35—H35	0.9500	N110—C110	1.323 (11)
C36—C37	1.471 (8)	N110—C114	1.356 (11)
C36—H36	0.9500	C110—C111	1.378 (13)
C37—C38	1.382 (8)	C110—H110	0.9500
C37—C41	1.387 (8)	C111—C112	1.361 (12)
C38—C39	1.388 (8)	C111—H111	0.9500
C38—H38	0.9500	C112—C113	1.381 (11)
C39—N31	1.336 (7)	C112—C115	1.495 (12)
C39—H39	0.9500	C113—C114	1.406 (12)
C40—N31	1.356 (7)	C113—H113	0.9500
C40—C41	1.372 (8)	C114—H114	0.9500
C40—H40	0.9500	C115—C116	1.260 (11)
C41—H41	0.9500	C115—H115	0.9500
N31—Co1^vi^	2.145 (5)	C116—C117	1.505 (12)
N50—C50	1.348 (7)	C116—H116	0.9500
N50—C54	1.351 (7)	C117—C121	1.367 (12)
C50—C51	1.367 (8)	C117—C118	1.383 (10)
C50—H50	0.9500	C118—C119	1.437 (11)
C51—C52	1.391 (8)	C118—H118	0.9500
C51—H51	0.9500	C119—N111	1.323 (11)
C52—C53	1.372 (8)	C119—H119	0.9500
C52—C55	1.453 (8)	C120—N111	1.349 (11)
C53—C54	1.391 (8)	C120—C121	1.365 (13)
C53—H53	0.9500	C120—H120	0.9500
C54—H54	0.9500	C121—H121	0.9500
C55—C56	1.328 (7)	O1—H1O1	0.8401
C55—H55	0.9500	O1—H2O1	0.8402
C56—C57	1.475 (8)	O2—H1O2	0.8401
C56—H56	0.9500	O2—H2O2	0.8399
C57—C58	1.379 (8)	O3—H1O3	0.8400
C57—C61	1.388 (8)	O3—H2O3	0.8401
C58—C59	1.402 (8)	O4—H1O4	0.8403
C58—H58	0.9500	O4—H2O4	0.8403
			
N1—Co1—N2	178.12 (15)	C55—C56—C57	125.4 (6)
N1—Co1—N31^i^	89.22 (17)	C55—C56—H56	117.3
N2—Co1—N31^i^	92.16 (19)	C57—C56—H56	117.3
N1—Co1—N30	88.41 (18)	C58—C57—C61	117.5 (5)
N2—Co1—N30	90.18 (19)	C58—C57—C56	123.0 (5)
N31^i^—Co1—N30	177.31 (18)	C61—C57—C56	119.6 (5)
N1—Co1—N10	90.58 (18)	C57—C58—C59	119.9 (5)
N2—Co1—N10	90.75 (19)	C57—C58—H58	120.1
N31^i^—Co1—N10	87.49 (16)	C59—C58—H58	120.1
N30—Co1—N10	93.80 (18)	N51—C59—C58	122.3 (5)
N1—Co1—N11^ii^	89.15 (18)	N51—C59—H59	118.8
N2—Co1—N11^ii^	89.56 (19)	C58—C59—H59	118.8
N31^i^—Co1—N11^ii^	90.45 (19)	N51—C60—C61	122.6 (5)
N30—Co1—N11^ii^	88.24 (15)	N51—C60—H60	118.7
N10—Co1—N11^ii^	177.9 (2)	C61—C60—H60	118.7
N4—Co2—N3	179.1 (2)	C60—C61—C57	120.0 (6)
N4—Co2—N50	91.03 (19)	C60—C61—H61	120.0
N3—Co2—N50	89.90 (18)	C57—C61—H61	120.0
N4—Co2—N51^iii^	89.52 (19)	C59—N51—C60	117.5 (5)
N3—Co2—N51^iii^	89.54 (18)	C59—N51—Co2^vii^	119.8 (4)
N50—Co2—N51^iii^	179.19 (18)	C60—N51—Co2^vii^	121.8 (4)
N4—Co2—N71^iv^	90.96 (19)	C74—N70—C70	118.2 (5)
N3—Co2—N71^iv^	88.98 (19)	C74—N70—Co2	121.5 (4)
N50—Co2—N71^iv^	92.88 (19)	C70—N70—Co2	119.9 (4)
N51^iii^—Co2—N71^iv^	87.70 (13)	N70—C70—C71	123.5 (5)
N4—Co2—N70	91.86 (19)	N70—C70—H70	118.3
N3—Co2—N70	88.19 (18)	C71—C70—H70	118.3
N50—Co2—N70	88.26 (13)	C70—C71—C72	119.3 (5)
N51^iii^—Co2—N70	91.14 (18)	C70—C71—H71	120.3
N71^iv^—Co2—N70	176.94 (16)	C72—C71—H71	120.3
C1—N1—Co1	169.4 (5)	C73—C72—C71	117.5 (5)
N1—C1—S1	179.4 (5)	C73—C72—C75	119.9 (6)
C2—N2—Co1	172.0 (5)	C71—C72—C75	122.5 (6)
N2—C2—S2	179.3 (6)	C72—C73—C74	119.5 (6)
C3—N3—Co2	174.2 (4)	C72—C73—H73	120.2
N3—C3—S3	179.0 (7)	C74—C73—H73	120.2
C4—N4—Co2	171.9 (4)	N70—C74—C73	121.9 (6)
N4—C4—S4	178.5 (6)	N70—C74—H74	119.1
C10—N10—C14	117.2 (5)	C73—C74—H74	119.1
C10—N10—Co1	120.8 (4)	C76—C75—C72	124.3 (7)
C14—N10—Co1	121.1 (4)	C76—C75—H75	117.8
N10—C10—C11	123.1 (6)	C72—C75—H75	117.8
N10—C10—H10	118.5	C75—C76—C77	124.0 (6)
C11—C10—H10	118.5	C75—C76—H76	118.0
C10—C11—C12	120.2 (5)	C77—C76—H76	118.0
C10—C11—H11	119.9	C81—C77—C78	118.3 (5)
C12—C11—H11	119.9	C81—C77—C76	121.4 (5)
C13—C12—C11	115.9 (5)	C78—C77—C76	120.3 (6)
C13—C12—C15	120.2 (6)	C77—C78—C79	118.9 (6)
C11—C12—C15	123.9 (6)	C77—C78—H78	120.6
C14—C13—C12	120.9 (6)	C79—C78—H78	120.6
C14—C13—H13	119.6	N71—C79—C78	122.7 (5)
C12—C13—H13	119.6	N71—C79—H79	118.7
N10—C14—C13	122.8 (5)	C78—C79—H79	118.7
N10—C14—H14	118.6	N71—C80—C81	122.4 (5)
C13—C14—H14	118.6	N71—C80—H80	118.8
C16—C15—C12	124.8 (6)	C81—C80—H80	118.8
C16—C15—H15	117.6	C80—C81—C77	119.5 (5)
C12—C15—H15	117.6	C80—C81—H81	120.2
C15—C16—C17	123.9 (6)	C77—C81—H81	120.2
C15—C16—H16	118.1	C79—N71—C80	118.2 (5)
C17—C16—H16	118.1	C79—N71—Co2^viii^	123.2 (4)
C21—C17—C18	116.4 (5)	C80—N71—Co2^viii^	118.2 (4)
C21—C17—C16	123.5 (5)	C94—N90—C90	117.2 (9)
C18—C17—C16	120.1 (5)	N90—C90—C91	121.9 (9)
C19—C18—C17	120.1 (5)	N90—C90—H90	119.0
C19—C18—H18	119.9	C91—C90—H90	119.0
C17—C18—H18	119.9	C92—C91—C90	119.0 (9)
N11—C19—C18	121.6 (5)	C92—C91—H91	120.5
N11—C19—H19	119.2	C90—C91—H91	120.5
C18—C19—H19	119.2	C93—C92—C91	117.7 (8)
N11—C20—C21	123.3 (5)	C93—C92—C95	119.5 (8)
N11—C20—H20	118.3	C91—C92—C95	122.7 (9)
C21—C20—H20	118.3	C92—C93—C94	118.9 (10)
C20—C21—C17	120.0 (5)	C92—C93—H93	120.5
C20—C21—H21	120.0	C94—C93—H93	120.5
C17—C21—H21	120.0	N90—C94—C93	125.1 (11)
C19—N11—C20	118.5 (5)	N90—C94—H94	117.4
C19—N11—Co1^v^	121.8 (4)	C93—C94—H94	117.4
C20—N11—Co1^v^	118.8 (4)	C96—C95—C92	127.3 (10)
C34—N30—C30	117.7 (5)	C96—C95—H95	116.3
C34—N30—Co1	124.7 (4)	C92—C95—H95	116.3
C30—N30—Co1	117.3 (4)	C95—C96—C97	124.9 (8)
N30—C30—C31	123.0 (5)	C95—C96—H96	117.5
N30—C30—H30	118.5	C97—C96—H96	117.5
C31—C30—H30	118.5	C98—C97—C101	117.8 (8)
C32—C31—C30	119.3 (5)	C98—C97—C96	117.2 (7)
C32—C31—H31	120.3	C101—C97—C96	125.0 (7)
C30—C31—H31	120.3	C97—C98—C99	118.2 (8)
C31—C32—C33	118.3 (5)	C97—C98—H98	120.9
C31—C32—C35	122.0 (5)	C99—C98—H98	120.9
C33—C32—C35	119.7 (5)	N91—C99—C98	124.5 (8)
C32—C33—C34	118.9 (5)	N91—C99—H99	117.8
C32—C33—H33	120.6	C98—C99—H99	117.8
C34—C33—H33	120.6	N91—C100—C101	122.7 (8)
N30—C34—C33	122.8 (5)	N91—C100—H100	118.6
N30—C34—H34	118.6	C101—C100—H100	118.6
C33—C34—H34	118.6	C100—C101—C97	120.1 (8)
C36—C35—C32	123.8 (6)	C100—C101—H101	120.0
C36—C35—H35	118.1	C97—C101—H101	120.0
C32—C35—H35	118.1	C99—N91—C100	116.7 (7)
C35—C36—C37	125.8 (6)	C110—N110—C114	116.1 (8)
C35—C36—H36	117.1	N110—C110—C111	124.8 (9)
C37—C36—H36	117.1	N110—C110—H110	117.6
C38—C37—C41	117.9 (5)	C111—C110—H110	117.6
C38—C37—C36	119.6 (5)	C112—C111—C110	119.7 (9)
C41—C37—C36	122.5 (5)	C112—C111—H111	120.2
C37—C38—C39	119.6 (5)	C110—C111—H111	120.2
C37—C38—H38	120.2	C111—C112—C113	117.7 (8)
C39—C38—H38	120.2	C111—C112—C115	118.6 (8)
N31—C39—C38	123.0 (5)	C113—C112—C115	123.7 (8)
N31—C39—H39	118.5	C112—C113—C114	119.5 (8)
C38—C39—H39	118.5	C112—C113—H113	120.2
N31—C40—C41	123.8 (5)	C114—C113—H113	120.2
N31—C40—H40	118.1	N110—C114—C113	122.1 (8)
C41—C40—H40	118.1	N110—C114—H114	118.9
C40—C41—C37	119.0 (5)	C113—C114—H114	118.9
C40—C41—H41	120.5	C116—C115—C112	125.7 (9)
C37—C41—H41	120.5	C116—C115—H115	117.2
C39—N31—C40	116.6 (5)	C112—C115—H115	117.2
C39—N31—Co1^vi^	121.4 (4)	C115—C116—C117	127.6 (10)
C40—N31—Co1^vi^	121.7 (4)	C115—C116—H116	116.2
C50—N50—C54	115.8 (5)	C117—C116—H116	116.2
C50—N50—Co2	123.5 (4)	C121—C117—C118	117.1 (8)
C54—N50—Co2	119.6 (4)	C121—C117—C116	120.5 (8)
N50—C50—C51	124.1 (5)	C118—C117—C116	122.4 (9)
N50—C50—H50	117.9	C117—C118—C119	118.7 (8)
C51—C50—H50	117.9	C117—C118—H118	120.7
C50—C51—C52	119.5 (6)	C119—C118—H118	120.7
C50—C51—H51	120.3	N111—C119—C118	122.7 (8)
C52—C51—H51	120.3	N111—C119—H119	118.6
C53—C52—C51	117.7 (5)	C118—C119—H119	118.6
C53—C52—C55	123.2 (6)	N111—C120—C121	123.3 (9)
C51—C52—C55	119.1 (6)	N111—C120—H120	118.3
C52—C53—C54	119.6 (5)	C121—C120—H120	118.3
C52—C53—H53	120.2	C120—C121—C117	121.4 (9)
C54—C53—H53	120.2	C120—C121—H121	119.3
N50—C54—C53	123.2 (6)	C117—C121—H121	119.3
N50—C54—H54	118.4	C119—N111—C120	116.8 (8)
C53—C54—H54	118.4	H1O1—O1—H2O1	113.1
C56—C55—C52	124.7 (6)	H1O2—O2—H2O2	101.6
C56—C55—H55	117.6	H1O3—O3—H2O3	111.6
C52—C55—H55	117.6	H1O4—O4—H2O4	94.8

Symmetry codes: (i) *x*+1/2, −*y*+1/2, *z*+1/2; (ii) *x*−1/2, −*y*+1/2, *z*+1/2; (iii) −*x*+3/2, *y*−1/2, *z*−1/2; (iv) −*x*+3/2, *y*−1/2, *z*+1/2; (v) *x*+1/2, −*y*+1/2, *z*−1/2; (vi) *x*−1/2, −*y*+1/2, *z*−1/2; (vii) −*x*+3/2, *y*+1/2, *z*+1/2; (viii) −*x*+3/2, *y*+1/2, *z*−1/2.

### Hydrogen-bond geometry (Å, º)

**Table:**

*D*—H···*A*	*D*—H	H···*A*	*D*···*A*	*D*—H···*A*
O1—H2*O*1···O3	0.84	2.00	2.825 (8)	169
O2—H2*O*2···O4	0.84	2.09	2.818 (8)	144
O3—H2*O*3···N91	0.84	2.15	2.933 (9)	156
O4—H1*O*4···N110	0.84	2.14	2.885 (9)	148
O1—H1*O*1···N90^ix^	0.84	2.07	2.877 (10)	160
O2—H1*O*2···N111^x^	0.84	2.03	2.858 (10)	170
O4—H2*O*4···O2^xi^	0.84	2.10	2.844 (8)	147

Symmetry codes: (ix) −*x*+2, −*y*+1, *z*−1; (x) *x*, *y*, *z*+1; (xi) −*x*+1, −*y*+1, *z*.
